# Supplementary material for: Screening for Parkinson’s disease using “computer vision”
Source: PLoS One. 2025 Aug 12;20(8):e0330373. doi: 10.1371/journal.pone.0330373 (PMC12342252; doi:10.1371/journal.pone.0330373)
Supplement: Attachment — Submitted filename: Response to Reviewers.docx [file pone.0330373.s001.docx]

TO:

John A. Thompson

Academic Editor
PLOS ONE

Re: PLOS ONE

PONE-D-24-40785

Using computer vision for screening parkinson’ disease

**Dear editors**

We are very grateful for the constructive reviewers’ comments, for the opportunity to improve upon our original manuscript, and for your consideration of this revised version. We have made changes to the original manuscript in order to address the reviewers’ concerns. These changes have been listed below. We have shared the code and data, which can be accessed via the URL provided in the Data availability section. All data has already de-identified.

We sincerely believe that the changes suggested by the reviewers have strengthened our study and will make for a greater impact of this article on the field of Parkinson’ disease. Thank you very much for your consideration of this updated version.

Sincerely,

Anupol Panitchote, MD.

(on behalf of all the authors)

**Response to reviewer comment**

**Reviewer #1:** The paper presents a study on detecting bradykinesia in Parkinson disease using computer vision applied to finger-tapping tests. The authors recorded 100 participants performing a 10-second finger-tapping test using a smartphone camera and extracted hand movement data with the Google MediaPipe Hands framework. The statistical analysis and machine learning classification results highlight the potential of non-contact, video-based PD screening. However, there are a few important aspects that can be addressed:

1. Validation of finger-tapping data extraction: the paper describes the use of MediaPipe Hands to estimate 3D landmarks but does not elaborate on how low-confidence landmarks or noisy data were handled. Details on confidence thresholds, data filtering or interpolation would enhance the reliability of the extracted data.

**Answer**: We add this paragraph to the Material and methods (Pose estimation and accuracy) part.

“MediaPipe Hands framework

The Google MediaPipe Hands (GMH) framework is a real-time hand-tracking system that leverages machine learning to detect and predict 21 three-dimensional (3D) landmarks for each hand. The framework consists of two submodules: palm detection and hand landmark detection. The palm detection submodule identifies a bounding box around the hand using a computationally efficient and robust model. This bounding box serves as the input for the hand landmark detection submodule. Within the detected bounding box, a regression model predicts the 3D coordinates of 21 key landmarks (e.g., fingertips, knuckles) in real time. The coordinates of each landmark, including those of the hand knuckles, consist of x, y, and z values, normalized to the range of 0.0 to 1.0 [16].

The GMH framework has been effectively validated for its accuracy in hand tracking and motion analysis. It has been applied in detecting and measuring resting tremors in Parkinson’s disease, with its performance confirmed against accelerometer-based systems The Google MediaPipe Hand (GMH) is a deep learning library utilized for hand tracking in a light-weight and portable machine learning pipeline. It consists of two sub-modules: palm detection and hand landmarks detection. GMH has been applied in detecting and measuring resting tremor in Parkinson’s disease, with its accuracy confirmed against an accelerometer [16, 17]. The validation study compares the GMH with a gold standard system using three dynamic hand exercises, namely hand open-closing, single finger tapping and multiple finger tapping, showing strong temporal and spectral consistency. It suggests a distance range of 60-100 cm, various motion speed (slow, normal, fast), and a lateral view angle [18].

Confidence Thresholds and Handling Noisy Data

The GMH framework utilizes a palm detection model as an initial step, specifically designed to exclude non-hand objects and reduce false positives. By incorporating bounding box localization and pre-trained models, the framework exhibits robustness across a variety of settings, including challenging conditions such as low light and partial occlusions. These features make it highly applicable to clinical and real-world scenarios, particularly in video-based motion analysis. The precision of the palm detector has been reported to range between 86.2% and 95.7% [19]. Additionally, the framework ensures stable pose predictions during continuous hand movements through the implementation of a temporal smoothing mechanism. However, a key limitation is that hand landmarks become unreliable when hands are partially obscured, and detection accuracy declines considerably in environments characterized by significant noise or dynamic activity. For stablizied GMH employs temporal filtering techniques to stabilize landmark detection across video frames. This helps to smooth out noise and jittering effects that might be exacerbated by tremors. We achieve this by adjusting the confidence thresholds used for both hand detection and landmark detection. In this study, we set the min_detection_confidence and min_tracking_confidence to the default value of 0.5.”

2. Feature interpretability and importance: while the authors use SHAP values to explain feature importance, a deeper exploration and explanation into the clinical significance of the top features would enhance the interpretability of the findings.

**Answer**: We add this paragraph to the discussion part.

“The difference in the number of taps between the right and left hands exhibits the highest SHAP value, underscoring its strong influence on the model's prediction. This metric likely captures asymmetry in finger-tapping performance, a hallmark motor symptom of Parkinson’s disease, particularly in its early stages. The second most influential feature, Tapping Score (Right), reflects finger-tapping performance, with reduced speed and irregularity being well-established indicators of bradykinesia. The third, Amplitude Decrement 1-10 (Left), measures the progressive decline in tapping amplitude over repeated tasks, a key characteristic of bradykinesia. These findings highlight the importance of assessing both the magnitude and variability of motor actions across hands. The use of computer vision to analyze subtle movement patterns enhances the sensitivity of these measurements, making it particularly valuable for detecting early bradykinesia, where traditional clinical assessments may lack precision. These results align with the study by Yang et al., which utilized three features—tapping rate and tapping amplitude variation—to train a classification model for assessing bradykinesia severity. However, one key difference is that their study included tapping freeze time as an additional variable.”

3. Handling of severe PD symptoms: The study briefly mentions that severe tremors might cause motion blur, leading to inaccurate measurements. However, no specific strategies to address this issue, such as filtering out affected. Additionally, it would be interesting if the authors check the performances and differences on the severe PD participants.

**Answer:** We add this paragraph to the material and methods (Confidence and Thresholds and handling noisy data) part.

…. For stablizied GMH employs temporal filtering techniques to stabilize landmark detection across video frames. This helps to smooth out noise and jittering effects that might be exacerbated by tremors. We achieve this by adjusting the confidence thresholds used for both hand detection and landmark detection. In this study, we set the min_detection_confidence and min_tracking_confidence to the default value of 0.5.

We add this paragraph to the Discussion part.

… Unfortunately, in our study, we didn't have patients with severe tremors; we tested on normal subjects. The GMH framework can detect tremors with an amplitude of less than 10 cm.

4. Machine learning validation: the study is limited to a single cohort, which raised concerns about the generalizability of the findings. Testing the model on cross validation or external datasets, or in different clinical settings would strengthen the applicability and reliability of the model.

**Answer:** We add this paragraph to the Material and methods part

Model development, validation and performance evaluation

We examined a diverse range of machine learning classifiers to identify the one with the highest categorical accuracy for the clinical diagnosis of Parkinson’s disease, specifically for patients with an MDS-UPDRS part III finger-tapping score of at least 1. The Python scikit-learn library, version 1.1.2[21], was utilized to train and test machine learning models in a Jupyter Notebook environment. The study involved the utilization and comparison of six machine learning algorithms, namely logistic regression classifier (LR), support vector machine classifier (SVM), random forest classifier (RF), decision tree (DT), light gradient boosting model (LGBM), and K-nearest neighbor classifier (KNN).

To ensure robust evaluation and prevent information leakage, we utilized a nested cross-validation framework. This approach involved two levels of cross-validation: an outer loop to assess model performance and an inner loop for hyperparameter tuning.

Outer Cross-Validation:

The outer loop employed a Stratified K-Fold Cross-Validation with 5 splits to ensure the class distribution of the target variable was preserved across training and test sets. For each fold, the dataset was split into distinct training and test sets, and the final evaluation metrics were derived from the model's performance on the unseen test data.

Inner Cross-Validation:

Within each training set generated by the outer loop, a 3-fold Stratified K-Fold Cross-Validation was conducted to perform hyperparameter tuning. Various methodologies were explored for tuning the hyperparameters of the six machine learning models, using GridSearchCV, to predict and analyze the presence of bradykinesia. The hyperparameter configuration yielding the highest accuracy in the inner loop was selected for training the model on the corresponding outer training set. A similar approach was applied for tuning the hyperparameters of other machine learning algorithms.

Model Evaluation and Metrics:

For each outer fold, the best model from the inner loop was used to predict probabilities and class labels on the outer test set. Performance was evaluated using multiple metrics, including accuracy, precision, recall, F1-score, and the area under the receiver operating characteristic curve (AUC). Additionally, the AUC was computed for both training and testing datasets to assess potential overfitting.

Confidence Interval Estimation:

To provide an estimate of the variability of the model's performance across folds, we calculated the mean, standard deviation, and 95% confidence intervals (CIs) for each evaluation metric. The CIs were derived using a t-distribution with degrees of freedom equal to the number of outer folds minus one. These CIs provide insight into the reliability of the reported performance metrics, considering variability across folds.

This nested cross-validation framework [23] applied across all six machine learning algorithms, ensures that the reported model performance is unbiased, reflective of real-world application, and provides robust insight into the effectiveness of different classifiers.

**Reviewer #2:** Peer review comments for the manuscript titled “Using computer vision for screening parkinson’ disease”

Thank you for the opportunity to review this manuscript. The study presents a method for utilizing computer vision to diagnose Parkinson’s Disease by analyzing videos of patients engaged in the finger-tapping task. However, there are a few concerns regarding the novel contribution to the field as well as ease-of-use by an interested third party. Below, I outline specific comments and suggestions for the authors to consider:

Major Comments

1. The manuscript claims novelty but does not adequately contextualize the study within the existing body of literature. Several relevant papers are not cited, which makes it difficult to evaluate how this work builds on or diverges from prior studies. Including a thorough literature review and discussing how this work advances the field would help clarify its unique contributions. Specifically, at lines 82:84 the authors state that “To the best of our knowledge, this study represents the first attempt to use computer vision techniques for screening Parkinson's disease by detecting bradykinesia in the finger tapping test for both hands.” However, none of the following four publications were cited, and yet they all describe methods using computer vision to diagnose Parkinson’s Disease by assessing the finger-tapping performance from both hands of their volunteers. To claim novelty, the authors of this manuscript should distinguish their work from these previously published methods.
   o Amprimo, G.; Rechichi, I.; Ferraris, C.; Olmo, G. Objective Assessment of the Finger Tapping Task in Parkinson’s Disease and Control Subjects using Azure Kinect and Machine Learning. In Proceedings of the 2023 IEEE 36th International Symposium on Computer-Based Medical Systems (CBMS), L’Aquila, Italy, 22–24 June 2023; pp. 640–645.
   o Wong, D.C.; Relton, S.D.; Fang, H.; Qhawaji, R.; Graham, C.D.; Alty, J.; Williams, S. Supervised Classification of Bradykinesia for Parkinson’s Disease Diagnosis from Smartphone Videos. In Proceedings of the 2019 IEEE 32nd International Symposium on Computer-Based Medical Systems (CBMS), Cordoba, Spain, 5–7 June 2019; pp. 32–37.
   o Li, Z.; Lu, K.; Cai, M.; Liu, X.; Wang, Y.; Yang, J. An Automatic Evaluation Method for Parkinson’s Dyskinesia Using Finger Tapping Video for Small Samples. J. Med. Biol. Eng. 2022, 42, 351–363.
   o Yang, N.; Liu, D.F.; Liu, T.; Han, T.; Zhang, P.; Xu, X.; Lou, S.; Liu, H.G.; Yang, A.C.; Dong, C.; et al. Automatic Detection Pipeline for Accessing the Motor Severity of Parkinson’s Disease in Finger Tapping and Postural Stability. IEEE Access 2022, 10, 66961–66973.

**Answer:** We add this paragraph to the discussion part.

The study employs computer vision to automatically classify bradykinesia by analyzing finger-tapping performance using the MDS-UPDRS score for multiclass prediction. A deep neural network (DNN) model achieved an average F1-score of 84–88%, as reported by Yang et al. Additionally, a convolutional neural network (CNN) model developed by Li et al. achieved an accuracy of 79.7% [31], aiming to automatically classify MDS-UPDRS scores. However, our study focuses on screening Parkinson’s disease by detecting bradykinesia and evaluating simultaneous finger tapping, assessing tapping performance in both hands, and analyzing inter-hand differences.The study by DC Wong, et al. also perform binary classficiation of presence of bradykinesia (UPDRS >1) using taping frequency, amplitude and tap-to-tap variability to predict bradykinesia on one hands, have a estimate accuracy 79% [32]. Amprimo et al. utilized the MediaPipe Hand framework and a specialized RGB-depth camera (Azure Kinect) to detect bradykinesia, achieving an accuracy of 94.4% and an F1 score of 98.4% in a leave-one-subject-out validation [33]. However, this approach requires a specialized RGB-depth camera for video recording.

2. Additionally, the "novel contribution" of the study is not clearly articulated. It would be beneficial for the authors to explicitly state what distinguishes their approach or findings from existing work.

**Answers**: We add this paragraph to the discussion part.

In this study, we introduced a novel approach utilizing computer vision techniques with MediaPipe hand to screen for Parkinson's disease by analyzing bradykinesia through finger tapping tests on smartphone video recordings. Uniquely, our study considers both hands simultaneously, contrasting with previous research that often focuses on a single hand measures. This dual-hand assessment enhances the detection of asymmetries and subtle bradykinesia, potentially improving early Parkinson's disease detection sensitivity. Notably, our machine learning model, developed from this data, has demonstrated strong predictability regarding bradykinesia, aiming to enhance diagnostic capabilities significantly.

3. The authors highlight that their method is easy to use, which is a commendable goal. However, the manuscript does not provide sufficient information on where to find the necessary resources (e.g., code, tools, datasets) or detailed instructions on how to use them. Including a link to the resources, accompanied by a brief user guide or example, would significantly enhance the usability and reproducibility of the method.

**Answer:** We add this paragraph to the discussion part.

We have also developed a web-based platform, pd.kku.ac.th/home, which allows users to screen for Parkinson’s disease by uploading a video. The website then processes the video, generates amplitude and time graphs, and provides results for bradykinesia detection.

We add this paragraph to the data availability part.

The code and data used for the statistical analysis of this work are available in the GitHub repository: https://github.com/Narongrit17/computer-vision-for-PD-screening.git

**Minor Comments**

1. Line 58 – The authors should briefly state why BRAIN TEST is inferior to their method.

**Answer:** We add this paragraph to the introduction part.

These include a computer software program called BRAIN TEST©, which assesses finger tapping speed and accuracy [4]. However, it requires a keyboard and may not be useful for individuals with severe hand dysfunction.

2. Line 100 – For clarity, please rework the sentence: “The modal clinician rating for each video was used for correlation with computer vision programmed.”

**Answer:** We add this paragraph to the materials and methods part.

For each video, the most common rating given by the clinician was used to measure how well it correlated with a computer vision programme assessment

3. I sympathize with the challenge of scientific writing in a non-native language, however the manuscript would benefit from careful editing, as multiple grammatical errors and typos were identifiable.

Suggestions for Improvement
• Include a comprehensive discussion of related work, with appropriate citations.
• Clearly articulate the novel contribution of the study in the introduction and discussion sections.
• Provide explicit guidance on accessing and using the method, such as a supplementary document or a dedicated section in the manuscript.
• Add more detailed explanations in the methods section to ensure reproducibility.
• Revise and refine the manuscript’s language for clarity and precision.

I appreciate the effort the authors have put into this manuscript and encourage them to address these points to maximize its impact. I look forward to seeing a revised version.

**Answer:** Thank you for your valuable feedback. I have carefully revised the manuscript to address all the points mentioned. I have made corrections and improvements in the Introduction, Materials and Methods, and Discussion sections, ensuring proper grammar and clarity. Please let me know if any further revisions are needed.

6. PLOS authors have the option to publish the peer review history of their article ([what does this mean?](https://www.google.com/url?q=https://track.editorialmanager.com/CL0/https:%252F%252Fjournals.plos.org%252Fplosone%252Fs%252Feditorial-and-peer-review-process%2523loc-peer-review-history/1/010f01944f2fc2e0-220e2ca8-36a2-4892-b7c1-dc8c7015ca3a-000000/BSwmtuNr1oVP6UVuofLZNNFRhri_rlHD_VAzB_5wZik%3D192&source=gmail-imap&ust=1737100121000000&usg=AOvVaw2cmWIUAgmq888opd6KQOai)). If published, this will include your full peer review and any attached files.


If you choose “no”, your identity will remain anonymous but your review may still be made public.
