# Supplementary material for: Screening for Parkinson’s disease using “computer vision”
Source: PLoS One. 2025 Aug 12;20(8):e0330373. doi: 10.1371/journal.pone.0330373 (PMC12342252; doi:10.1371/journal.pone.0330373)
Supplement: Attachment — Submitted filename: Response to Reviewers .docx [file pone.0330373.s002.docx]

TO:

John A. Thompson

Academic Editor
PLOS ONE

Re: PLOS ONE

PONE-D-24-40785

**Screening for Parkinson’s disease using “computer vision”**

**Dear editors**

We have appreciation to the reviewers for their thoughtful and constructive feedback, which has greatly contributed to improving the quality of our manuscript. We have carefully addressed all comments, and the corresponding revisions are outlined below.

To improve the clarity and precision of reporting, the revised manuscript has undergone thorough language editing by a native English speaker. Moreover, transparency and reproducibility of our work, we have shared the complete de-identified dataset and annotated analysis code on Figshare, accessible via the DOI provided in the Data Availability section. Furthermore, we have developed a user-friendly online tool that allows others to interactively explore the model and apply it to their own data. We believe this open-access approach will facilitate broader use and validation of our findings.

We are confident that these improvements have strengthened the manuscript and increased its potential impact within the field.

Sincerely,

Anupol Panitchote, MD.

(on behalf of all the authors)

**Review Comments to the Author**

Please use the space provided to explain your answers to the questions above. You may also include additional comments for the author, including concerns about dual publication, research ethics, or publication ethics. (Please upload your review as an attachment if it exceeds 20,000 characters)

**Reviewer #1:** The authors have addressed the previous concerns raised in the first review round. The expanded explanation of MediaPipe Hands, implementation of SHAP interpretation, and description of the nested cross-validation framework are appreciated.

**Reviewer #2:** I appreciate the authors’ time and effort in revising the manuscript and providing detailed responses to the initial round of feedback. However, after reviewing the revised version and accompanying materials, I believe that several critical issues remain unresolved, and I must recommend rejection at this time.

1. Clarity of Writing

Unfortunately, the clarity of the manuscript remains a significant concern. Many sentences are difficult to interpret, and in several instances, the intended meaning is unclear. While it is entirely understandable that English may not be the authors’ first language, the current level of writing poses a barrier to understanding the core ideas and contributions of the work. I encourage the authors to seek assistance from a fluent English speaker or professional editing service to improve the readability of the manuscript before resubmission elsewhere.

**Answer**: We sincerely appreciate the reviewer’s feedback regarding the clarity of the manuscript. To address this concern, we have sought assistance from Mr. Bryan Roderick Hamman, a native English speaker and a member of the Council of Science Editors. He provided support with the English-language presentation of the manuscript through Khon Kaen University’s Publication Clinic, Thailand. We believe the revised version is now significantly clearer and more readable.

2. Code Availability and Reproducibility

In response to requests for code sharing, the authors provided a Jupyter notebook and a rudimentary readme document. However, the notebook is not sufficiently structured for others to understand or reproduce the analysis. A well-organized and clearly annotated codebase would be more appropriate and aligned with the journal’s expectations for open and reproducible science.

**Answer:** We have improved the structure and clarity of our shared resources to better align with the journal’s expectations for open and reproducible science. Specifically:

- We now provide a detailed README file outlining the step-by-step workflow.
- A Jupyter Notebook with clearer annotations and logical structure is included.
- An environment.yml file is provided to facilitate the setup of the required computational environment.

All code, data, and supporting files necessary to reproduce our results are publicly available on Figshare at the following links:

- README: <https://doi.org/10.6084/m9.figshare.28883756.v2>
- Environment setup file: <https://doi.org/10.6084/m9.figshare.28931441.v1>
- Jupyter Notebook: <https://doi.org/10.6084/m9.figshare.28883624.v2>
- Dataset (Parkinson_data.csv): <https://doi.org/10.6084/m9.figshare.28883489.v1>
- Example video (finger tapping): <https://doi.org/10.6084/m9.figshare.28930706.v1>
- Example extracted data (1 case): <https://doi.org/10.6084/m9.figshare.28930694.v1>
- SVC model file: https://doi.org/10.6084/m9.figshare.28883657.v2

We hope these revisions adequately address your concerns and make the study fully reproducible.

3. Functionality of Online Tool

The authors mention the development of an online resource intended to make their work more accessible. However, at the time of review, the website does not appear to function as intended. I encourage the authors to ensure that any publicly accessible resources are fully operational before including them in future submissions.

**Answer:** We have identified and resolved the issue that affected the website’s performance during the review period. The online tool is now fully operational and accessible at: KKU Parkinson Detection (https://pd.kku.ac.th/home). We have verified that all core functionalities are working as intended, and we will continue to monitor and maintain the platform to ensure its reliability.
