# Supplementary material for: Screening for Parkinson’s disease using “computer vision”
Source: PLoS One. 2025 Aug 12;20(8):e0330373. doi: 10.1371/journal.pone.0330373 (PMC12342252; doi:10.1371/journal.pone.0330373)
Supplement: Attachment — Submitted filename: Response_to_Reviewers_auresp_3.docx [file pone.0330373.s003.docx]

TO:

John A. Thompson

Academic Editor
PLOS ONE

Re: PLOS ONE

PONE-D-24-40785

**Screening for Parkinson’s disease using “computer vision”**

**Dear editors**

We have appreciation to the reviewers for their thoughtful and constructive feedback, which has greatly contributed to improving the quality of our manuscript. We have carefully addressed all the comments, and the corresponding revisions are outlined below. We have also carefully checked the English grammar in response to the reviewers’ suggestions. Furthermore, we have corrected the website and revised some of the code, as requested.

For the references, we did not cite the retracted publication, and we have also updated Reference 12 with the final published version of the article.

We are confident that these improvements have strengthened the manuscript and increased its potential impact within the field.

Sincerely,

Anupol Panitchote, MD.

(on behalf of all the authors)

Reviewer #2: The authors should be commended for the effort to create a website that detects bradykinesia in a video uploaded from mobile devices. However, there are a couple of problems that need to be addressed.

1) The website only works if all fingers are visible in every frame. If an individual clicks record, sets the phone down, and then starts tapping, the video is not usable and hangs without warning or explanation. The authors should either add more detail to the instructions or, preferably, edit the software to ignore early frames in which both hands may not be visible. If an error does occur, the website should alert the user of the error instead of hanging indefinitely.

Answer: We have addressed this by modifying the code to be more robust. The application will now automatically detect when finger tapping begins within the video. It will then proceed to process the video and generate the graph specifically from the point where the finger tapping action is identified. This ensures that only the relevant segments of the video are analyzed, and it will also ensure a total duration of at least 10 seconds of actual tapping is used for analysis. (This addresses the scenario where a user might start recording, setting the phone down, and then begin tapping).

The images provided illustrate this improvement:

Original scenario (left graphs): Shows erratic data before the tapping truly begins.

Improved scenario (right graphs): Demonstrates that the analysis now accurately starts from the moment finger tapping is detected, providing cleaner and more reliable data, ensuring 10 seconds of tapping is analyzed.

This adjustment will prevent the website from hanging and will provide a more seamless and user-friendly experience, as it no longer requires users to ensure fingers are visible from the very beginning of the recording.

After downloading the .mov file, the preview is currently not supported. However, the website is able to detect the .mov file correctly. We are working on resolving this issue. The graph, however, is displaying properly.

2) Considering the popularity of the iPhone and other Apple products, the authors should include .mov as an acceptable video file, since .mov is the native file extension on Apple products.

Answer: We have updated our website and application requirements to include .mov as an acceptable video file format. The revised text is as follows: “Please upload your video to the application. It must be in .mp4 or .mov format and recorded at 30 frames per second (fps).”

3) According to your Materials and Methods section, the healthy participants in your study were excluded if they had bradykinesia, motor weakness, joint stiffness, joint deformity, or inability to perform the finger-tapping test due to arthritis or pain. The website should alert users that a false-positive diagnosis could result if they have any of these conditions.
**Answer:** We have updated the website to include a Caution message that addresses this concern, alongside the existing Requirements:

**Requirement:** Video should be at least 10 seconds long (processing takes approximately 30 seconds). The video must be in .mp4 or .mov format, and the duration of finger tapping should be at least 10 seconds.

**Caution:** If the test subject has muscle weakness, joint deformity, joint stiffness, or is unable to perform the finger-tapping test due to arthritis or pain, it may lead to a misinterpretation as bradykinesia, even though the person does not have Parkinson’s disease (false positive).

The authors should be commended for their effort editing the manuscript for clarity. The following list includes the remaining errors which need to be corrected.
line 38 "features was" should be "features were"
line 57 "patient" should be "patients"
line 76 "forassessing" should be "for assessing"
line 93 "perfrom" should be "perform"
line 95 "Four neurologists were rate 100 videos" should be "Four neurologists rated 100 videos"
line 133 "implementating" should be "implementing"
line 136 "stablizied" should be "stabilized"
line 142 insert a space before the bracket
line 177 insert a space after the comma
line 178 insert a space before parenthesis
line 179 "enviroment" should be "environment"
line 160 The text should be "within a 10-s window"
line 196 "for train the model" should be "for training the model"
line 197 "tune" should be "tuning"
line 214 insert a space before the bracket
line 222 "fail" should be "failure"
line 228 insert a space before the parenthesis
line 231 "slightmild" should be either "slight" or "mild"
line 254 "choice for for this" should be "choice for this"
line 257 replace "for the" with "to"
line 266 insert a space after "values"
line 281 insert a space after the period
line 285 insert a space before "[12]"
line 295 insert a space after the period
line 312 "classficiation" should be "classification"
line 319 "are various of" should be "are various"
line 329 "are" should be "is"
line 330 "it is can utilize" should be "it can utilize"
line 349 "tremors amplitude are" should be "tremor amplitude is"
line 352 insert a space after the period
line 366 "supportedby" should be "supported by"
In general, there should be a space before all brackets and parentheses, and there should be a space after all commas.

Answer: We have checked the grammar and typos.
